# TargetCall: eliminating the wasted computation in basecalling via pre-basecalling filtering

**DOI:** 10.3389/fgene.2024.1429306

**Published:** 2024-10-28

**Authors:** Meryem Banu Cavlak, Gagandeep Singh, Mohammed Alser, Can Firtina, Joël Lindegger, Mohammad Sadrosadati, Nika Mansouri Ghiasi, Can Alkan, Onur Mutlu

**Affiliations:** ^1^ SAFARI Research Group, Department of Information Technology and Electrical Engineering, ETH Zurich, Zurich, Switzerland; ^2^ Department of Computer Engineering, Bilkent University, Ankara, Türkiye

**Keywords:** nanopore sequencing, basecalling, deep learning, filtering, efficiency

## Abstract

Basecalling is an essential step in nanopore sequencing analysis where the raw signals of nanopore sequencers are converted into nucleotide sequences, that is, reads. State-of-the-art basecallers use complex deep learning models to achieve high basecalling accuracy. This makes basecalling computationally inefficient and memory-hungry, bottlenecking the entire genome analysis pipeline. However, for many applications, most reads do not match the reference genome of interest (i.e., target reference) and thus are discarded in later steps in the genomics pipeline, wasting the basecalling computation. To overcome this issue, we propose TargetCall, the first pre-basecalling filter to eliminate the wasted computation in basecalling. TargetCall’s key idea is to discard reads that will not match the target reference (i.e., off-target reads) prior to basecalling. TargetCall consists of two main components: (1) LightCall, a lightweight neural network basecaller that produces noisy reads, and (2) Similarity Check, which labels each of these noisy reads as on-target or off-target by matching them to the target reference. Our thorough experimental evaluations show that TargetCall 1) improves the end-to-end basecalling runtime performance of the state-of-the-art basecaller by 3.31× while maintaining high (98.88%) recall in keeping on-target reads, 2) maintains high accuracy in downstream analysis, and 3) achieves better runtime performance, throughput, recall, precision, and generality than prior works. TargetCall is available at https://github.com/CMU-SAFARI/TargetCall.

## 1 Introduction

Genome sequencing, which determines the nucleotide sequence of an organism’s genome, plays a pivotal role in enabling many medical and scientific advancements (Alkan et al., 2009; Ashley, 2016; Chin et al., 2011; Ellegren, 2014; Alvarez-Cubero et al., 2017). Modern sequencing technologies produce increasingly large amounts of genomic data at low cost (Wang et al., 2021). Leveraging this genomic data requires fast, efficient, and accurate analysis tools.

Current sequencing machines are unable to determine an organism’s genome as a single contiguous sequence (Alser et al., 2021). Instead, they sequence fragments of a genome, called *reads*. The length of the reads depends on the sequencing technology and significantly affects the performance (i.e., speed or runtime) and accuracy of genome analysis. The use of *long reads* can provide higher accuracy and performance on many genome analysis steps (Treangen and Salzberg, 2011; Firtina and Alkan, 2016; Alkan et al., 2010; Lu et al., 2016; Magi et al., 2018; Firtina et al., 2023b).

Nanopore sequencing technology is one of the most prominent and widely used long read sequencing technologies (Wang et al., 2021; Branton et al., 2008; Gong et al., 2019; Jain et al., 2018; Logsdon et al., 2020; Amarasinghe et al., 2020). Nanopore sequencing relies on measuring the change in the electrical current when a nucleic acid molecule (DNA or RNA) passes through a pore of nanometer size (Zhang et al. 2021a). The measurement of the electrical current, called a *raw signal*, is converted to a nucleotide sequence, called a *read*, with a step called *basecalling* (Wang et al., 2021; Alser et al., 2021; Wick et al., 2019; Pagès-Gallego and de Ridder, 2023; Alser et al., 2022; Wan et al., 2021). Basecalling commonly uses computationally expensive deep neural network (DNN)-based architectures to achieve high basecalling accuracy (Senol Cali et al., 2019; Rang et al., 2018; Singh et al., 2024), which makes basecalling a computational bottleneck for genome analysis that consumes up to 84.2% of total execution time in the genome analysis pipeline (Bowden et al., 2019). However, the majority of this computation is wasted for genome sequencing applications that do not require most of the basecalled reads. For example, in SARS-CoV-2 genome assembly, 96% of the total runtime is spent on basecalling, even though ≥ 99% of the basecalled reads are not required after basecalling because they are not coming from the reference genome that is targeted by the application (Dunn et al., 2021). Therefore, it is important to eliminate wasted computation in basecalling.

Our goal in this work is to eliminate the wasted computation when basecalling the entire read, while maintaining high accuracy and applicability to a wide range of genome sequencing applications. To this end, we propose TargetCall, the *first* pre-basecalling filter. TargetCall is based on the key observation that the typical reason for discarding basecalled reads is that they do not match some *target reference* (e.g., a reference genome of interest) (Grumaz et al., 2016; Dunn et al., 2021). We call these *off-target* reads. Our key idea is to filter out off-target reads before basecalling by analyzing the *entire* read with a highly accurate and high-performance *pre-basecalling filter* to eliminate the wasted computation in basecalling off-target reads.

Prior works in targeted sequencing (Kovaka et al., 2021; Zhang et al., 2021a; Dunn et al., 2021; Bao et al., 2021; Firtina et al., 2023a; Lindegger et al., 2023; Firtina et al., 2024) propose *adaptive sampling* techniques to discard off-target reads during sequencing to better utilize sequencers. Sequencers provided by Oxford Nanopore Technologies (ONT) can enable adaptive sampling with a feature known as *Read Until* (Kovaka et al., 2021; Loose et al., 2016). ONT sequencers that support Read Until can selectively remove a read from the nanopore while the read is being sequenced. This requires a method of identifying which reads are off-target for further downstream analysis to decide which reads to remove from the nanopore. The state-of-the-art adaptive sampling methods can be classified into three groups based on the methodology used to label the read. The first group converts the target reference into a reference raw signal and performs raw signal-level alignment (Zhang et al., 2021a; Dunn et al., 2021; Loose et al., 2016). The second group generates noisy sequence representations of the raw signal to compare them with the target reference (Kovaka et al., 2021; Payne et al., 2020). The third group of works utilizes neural network classifiers to label the sequences (Bao et al., 2021; Noordijk et al., 2023). We provide a detailed background on different adaptive sampling approaches in Section 1 of the Supplementary Material.

Even though the labeling techniques of adaptive sampling can be repurposed for pre-basecalling filtering, the adaptive sampling problem is different from pre-basecalling filtering for three main reasons. First, in adaptive sampling, reads must be labeled during sequencing, requiring only the initial portion of the raw signal to classify reads as off-target or on-target. Analyzing a sub-region or raw signals in adaptive sampling methods often leads to low recall (77.5%−90.40%) (Kovaka et al., 2021; Zhang et al., 2021a) or poor basecalling Zhang et al., 2021a; Payne et al., 2020), meaning they can falsely reject many on-target reads. In contrast, a pre-basecalling filter can utilize the entire raw signal after the read is fully sequenced, enabling more accurate classification. Second, adaptive sampling has practical limitations, such as the risk of nanopores becoming blocked after a few seconds of sequencing (Munro et al., 2024), which limits the effectiveness of read ejection. A pre-basecalling filter addresses these limitations by processing the whole signal of all reads, even when adaptive sampling is not feasible. Third, some adaptive sampling methods require re-training classifiers for each different application and target reference (Bao et al., 2021), while a pre-basecalling filter can be applied without requiring re-training, making it more flexible across different use cases. We conclude that pre-basecalling filtering is orthogonal to adaptive sampling and can complement it. Even when adaptive sampling is used to reject reads early, any remaining reads still need to be basecalled, and a pre-basecalling filter can further improve accuracy and efficiency by processing the entire signal of these remaining reads. This makes the pre-basecalling filter a versatile solution that can be applied both independently and in conjunction with adaptive sampling approaches. TargetCall aims to overcome the challenges of state-of-the-art methods by utilizing the entire raw signal for classification, making it a widely applicable solution.

TargetCall consists of two main components: 1) *LightCall*, a lightweight basecaller with a simple neural network model that outputs erroneous (i.e., noisy) reads with high performance and 2) *Similarity Check* to compute the similarity of the noisy read to the target reference where the similarity is determined by the conventional read mapping pipeline. LightCall’s model is 33.31× smaller than the state-of-the-art basecaller, Bonito (2022). This reduction in the model improves the basecalling speed substantially with a small (4.85%) reduction in basecalling accuracy. Although reducing the basecalling accuracy might cause LightCall to be not applicable to some of the downstream analyses that require high basecalling accuracy (Frei et al., 2021), it is sufficient for Similarity Check to perform pre-basecalling filtering. We use the state-of-the-art read mapper minimap2 (Li, 2018) for Similarity Check. TargetCall overcomes all three limitations of prior methods. First, Similarity Check’s high accuracy enables TargetCall’s accuracy to be significantly higher than prior adaptive sampling approaches. Second, LightCall’s performance is independent of the target reference size, which enables TargetCall to be applicable to target reference sizes for which prior works were inapplicable. Third, unlike prior approaches that require re-training the network for each application and target reference, LightCall does not need to be re-trained.

### 1.1 Key results

We evaluate the performance and accuracy impact of TargetCall on the state-of-the-art basecaller by Bonito (2022) and compare TargetCall with two state-of-the-art adaptive sampling methods, UNCALLED (Kovaka et al., 2021) and Sigmap (Zhang et al., 2021a), repurposed as pre-basecalling filters. TargetCall 1) improves the end-to-end runtime performance (i.e., runtime of all the steps, including basecalling and read mapping, used in an analysis) by 3.31× over Bonito, 2) precisely filters out 94.71% of the off-target reads, and 3) maintains high recall in keeping on-target reads with 98.88% recall. We show that TargetCall provides high accuracy in a specific downstream analysis that aims to estimate the relative abundance (RA) of organisms in a given sample even after losing 1.12% of on-target reads. We demonstrate that TargetCall improves 1) runtime performance by 1.46× / 9.72× , 2) throughput by 1124.03× / 42.08× , 3) recall by + 3.09% /+ 23.15% , and 4) precision by + 58.48% /+ 62.31% over prior works UNCALLED/Sigmap while requiring much less peak memory (on average 5.76× ) and maintaining scalability to longer target references.

This article makes the following contributions:

• We introduce the problem of pre-basecalling filtering that aims to classify reads utilizing the entire raw signal information.

• We introduce the first pre-basecalling filter that eliminates the wasted computation in basecalling by leveraging the fact that most reads are discarded after basecalling.

• We propose LightCall, a lightweight neural network model that significantly increases the performance of basecalling with minor reductions in basecalling accuracy.

• TargetCall provides larger runtime performance and accuracy benefits than the state-of-the-art adaptive sampling works for basecalling.

• To aid research and reproducibility, we freely open source our implementation of TargetCall at https://github.com/CMU-SAFARI/TargetCall.

## 2 Materials and methods

Our goal in this work is to eliminate the wasted computation in basecalling using an accurate pre-basecalling filtering technique. To this end, we propose TargetCall, which can perform pre-basecalling filtering in *all* genome sequencing applications accurately and efficiently without any additional overhead. To our knowledge, TargetCall is the first pre-basecalling filter that is applicable to a wide range of use cases and makes use of entire raw signal information to classify nanopore raw signals. TargetCall’s key idea is to quickly filter out off-target reads (i.e., reads that are dissimilar to the *target reference*) before the basecalling step to eliminate the wasted computation in basecalling. We present the high-level overview of TargetCall in Section 2.1 and explain its components in Sections 2.2, 2.3.

### 2.1 High level overview

**FIGURE 1:**
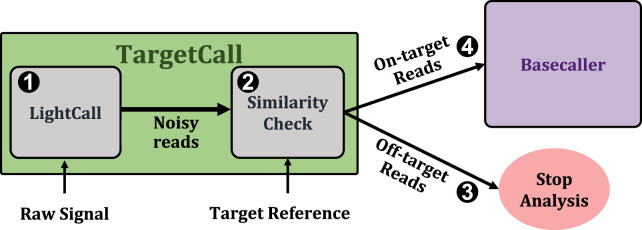
High-level overview of TargetCall.

Figure 1 shows TargetCall’s workflow. First, TargetCall performs noisy basecalling on the raw signal using LightCall (1). The output sequence of LightCall is highly accurate but erroneous compared to the reads basecalled using state-of-the-art basecallers. Second, Similarity Check compares the noisy read of LightCall to the target reference to label the read as an on-target or off-target read (2). TargetCall stops the analysis of off-target reads by removing them from the pipeline (3), whereas the analysis of the on-target reads continues with basecalling following the usual genomics pipeline to maintain basecalling accuracy^1^ (4).

The choice of the target reference depends on the specific genome sequencing application. The size of the target reference is a major constraint in most prior works, limiting the generality of the prior approaches. We design TargetCall such that its runtime performance scales well with the size of the target reference so that it is applicable to any genome sequencing application. We achieve our design goal because 1) the performance of LightCall is independent of the size of the target reference, and 2) the performance of the Similarity Check module scales well with the increasing target reference size.

### 2.2 LightCall

LightCall, the first component of TargetCall, is a lightweight neural network-based basecaller that produces noisy reads. Although LightCall is not designed to be as accurate as the state-of-the-art basecallers, its combination with Similarity Check is effective in determining if the read is an on-target read with respect to the target reference. We develop LightCall by modifying the state-of-the-art basecaller Bonito’s architecture in three ways: 1) reducing the channel sizes of convolution layers, 2) removing the skip connections, and 3) reducing the number of basic convolution blocks. Prior work (Singh et al., 2024) shows that Bonito’s model is over-provisioned, and we can maintain very high accuracy with reduced model sizes. Following prior work’s insight, we generate different neural network models by pruning the channel sizes of convolution and convolution blocks. The specific LightCall configurations are tested and designed based on our intuition. We select the neural network architecture for LightCall that balances basecalling accuracy with the pre-basecalling filtering performance.

**FIGURE 2:**
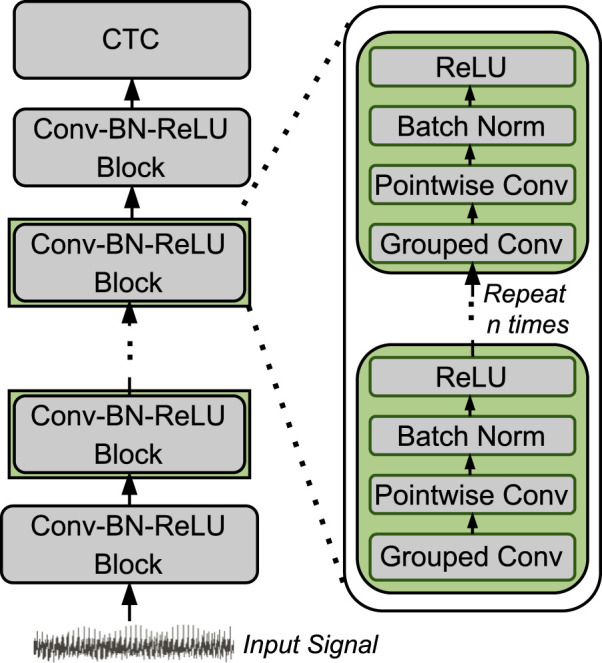
Overview of LightCall architecture.

Figure 2 shows the architecture of LightCall. Each block consists of grouped 1-dimensional convolution and pointwise 1-dimensional convolution. The convolution operation is followed by batch normalization (Batch Norm) (Ioffe and Szegedy, 2015) and a rectified linear unit (ReLU) (Agarap, 2018) activation function. The final output is passed through a connectionist temporal classification (CTC) (Graves et al., 2006) layer to produce the decoded sequence of nucleotides (A, C, G, and T). The CTC layer acts as the loss function by providing the correct alignment between the input and the output sequence. The CTC loss allows the model to handle the variable-length sequences by aligning the model’s output to the target sequence while ignoring the blank or padding symbols. Our LightCall architecture is composed of 18 convolution blocks containing ∼ 292,000 model parameters ( ∼33.35× fewer parameters than Bonito).

Modern deep learning-based basecallers (Singh et al., 2024; Wick et al., 2019; Neumann et al., 2022; Konishi et al., 2021; Xu et al., 2021; Lou et al., 2020; Perešíni et al., 2021; Pagès-Gallego and de Ridder, 2023; Zhang et al., 2021b) incorporate skip connections to help mitigate the vanishing gradient and saturation problems (V and Kiran, 2022). Removing skip connections has a higher impact on basecalling accuracy. However, adding skip connections introduces the following three issues for performance (Shahroodi et al., 2023; Singh et al., 2024). First, the skip connections increase the data lifetime. The layers whose activations are reused in subsequent layers must wait for this activation reuse (or buffer the activations in memory) before accepting new input and continuing to compute. This leads to high resource and storage requirements due to data duplication. Second, skip connections introduce irregularity in neural network architecture as these connections span non-adjacent layers. Third, skip connections require additional computation to adjust the channel size to match the channel size at the non-consecutive layer’s input. Therefore, we remove the skip connections, as we can tolerate a lower LightCall accuracy to improve the performance of TargetCall.

LightCall works by splitting a long read in raw signal format (e.g., millions of samples) into multiple smaller chunks (e.g., thousands of samples per chunk) and basecalling these chunks. The CTC layer assigns a probability for all possible labels (i.e., A, C, G, and T) at each sequence position for a chunk. The label with the highest probability is selected as the final output for a sequence position. LightCall merges the outputs of each position to produce the basecalled chunk and merges the basecalled chunks to output the basecalled read. Because LightCall’s algorithm is independent of the target reference, LightCall’s performance does not depend on the target reference length.

### 2.3 Similarity check

After LightCall outputs the noisy read that approximately represents the raw signal, Similarity Check compares the noisy read to the target reference. For this task, we use a procedure common in genome analysis known as sequence alignment. Sequence alignment computes the similarity between a read and a reference. To provide a scalable and fast solution for Similarity Check, we use minimap2, a well-optimized sequence aligner designed by Li (2018). Similarity Check labels a read that is similar to the target reference, that is, a read that maps to the target reference as on-target. With the efficient index structure of minimap2, the performance of sequence alignment is almost independent of the length of the target reference genome. Hence, TargetCall is scalable to large target reference genomes of Gbp (i.e., Giga base pair) length.

The accuracy of the computed sequence alignments is not high enough to represent the true alignment between the read and the target reference because the reads computed by LightCall are noisy. However, the labeling accuracy of Similarity Check for determining the on-target/off-target reads is high enough to provide recall up to 99.45% in filtering reads. The minor inaccuracy of Similarity Check can be compensated with the high sequencing depth-of-coverage, the average number of reads that align to a genomic region, required for confident genome sequence analysis (Quail et al., 2012; Levy and Myers, 2016; Hu et al., 2021; Sims et al., 2014).

## 3 Results

### 3.1 Experimental setup

#### 3.1.1 Evaluated use cases

To show the applicability of TargetCall, we evaluate it on use cases with varying target reference sizes without compromising the accuracy. We describe three use cases we use to evaluate TargetCall: (1) COVID detection, (2) sepsis detection, and (3) viral detection. All three use cases contain a significant fraction of off-target reads that are eliminated using TargetCall to show the benefits of pre-basecalling filtering.

#### 3.1.2 COVID detection

The first use case aims to accept reads coming from a small target reference. We choose SARS-CoV-2 detection as a sample biological application where the goal is to detect the reads coming from a SARS-CoV-2 reference genome ( ∼ 30 Kbp) from a sample taken from a human and filter out the human reads in the sample as performed by Dunn et al. (2021) and Geoghegan et al. (2021).

#### 3.1.3 Sepsis detection

The second use case aims to filter reads when the target reference is large ( ∼ 3 Gbp) to show that TargetCall is applicable to use cases with large target references. We choose sepsis detection, as described by Grumaz et al. (2016), Celik et al. (2022), and Sands et al. (2021), as a sample biological application where the goal is to delete the human reads from a human sample. Because the bacteria causing the disease are unknown, we cannot search for reads coming from a specific bacterial target. Instead, we apply TargetCall to filter out reads similar to the target reference.

#### 3.1.4 Viral detection

The third use case aims to filter reads when the target reference contains a collection of reference genomes to show that TargetCall can correctly filter reads when the sample and the target reference have a wide variety of species. This is to test the specificity of TargetCall in filtering reads when the on-target and off-target reads resemble each other more than in previous use cases. We choose disease-causing viral read detection as a sample biological application where the goal is to detect the viral reads from a metagenomic sample of bacterial and viral reads, and the target reference contains a collection of viral reference genomes, as demonstrated by Mokili et al. (2012).

#### 3.1.5 Evaluation system

We use NVIDIA TITAN V to train and evaluate the LightCall and Bonito baseline. For our evaluations, we increase the batch size maximally such that the entire GPU memory is occupied (Sections 3.2–3.5). We use NVIDIA A100 to evaluate TargetCall against prior work. We evaluate UNCALLED and Sigmap on a high-end server (AMD EPYC 7742 CPU with 1 TB DDR4 DRAM). For our evaluations, we optimize the number of threads (128) for UNCALLED/Sigmap and the batch size (128) for TargetCall such that all tools have the minimum execution time (Section 3.6). We use the state-of-the-art read mapper, minimap2 (Li, 2018), for the Similarity Check module of TargetCall with -a and -x map-ont flags. The -a flag is used to compute sequence alignments, and -x map-ont is used to configure minimap2 parameters for ONT data.

#### 3.1.6 Training setting

We use the publicly available ONT dataset, Bonito (2022), sequenced using MinION Flow Cell (R9.4.1) (ONT, 2022) for the training and validation. The neural network weights are updated using the Adam optimizer (Kingma and Ba, 2014) with a learning rate of 2 $e^{-3}$ , a beta value of 0.999, a weight decay of 0.01, and an epsilon of 1 $e^{-8}$ .

#### 3.1.7 Baseline techniques

We evaluate TargetCall’s runtime performance and accuracy as a pre-basecalling filter by integrating it as a pre-basecalling filter to Bonito (2022), which is one of the official basecalling tools developed by ONT. In Section 3.6, we evaluate two state-of-the-art, non-machine learning-based adaptive sampling methods, UNCALLED (Kovaka et al., 2021) and Sigmap (Zhang et al., 2021a), repurposed as pre-basecalling filters to compare against TargetCall. We repurposed UNCALLED and Sigmap by using their classification methods to classify the reads as on-target/off-target before the basecalling step without any change in their implementations. We identify the ground truth on-target and off-target by basecalling the reads using a high accuracy Bonito model followed by performing minimap2. We do not evaluate TargetCall against other adaptive sampling methods, such as SquiggleNet (Bao et al., 2021), that cannot be trivially used as pre-basecalling filters.

#### 3.1.8 LightCall configurations evaluated

To determine the final architecture of TargetCall, we test 5 different LightCall configurations. Table 1 lists the LightCall configurations evaluated.

**TABLE 1:** Different LightCall configurations.

Model name	Number of parameters (K)	Model size (MB)
Bonito	9,739	37.14
$LC_{\mathrm{Main\times 2}}$	565	2.16
$LC_{\mathrm{Main}}$	292	1.11
$LC_{\mathrm{Main∕2}}$	146	0.55
$LC_{\mathrm{Main∕4}}$	52	0.19
$LC_{\mathrm{Main∕8}}$	21	0.07

#### 3.1.9 Evaluated datasets

We sampled 287,767 reads from prior work (Zook et al., 2019; cad, 2020; Wick et al., 2019) and simulated 35,000 reads using DeepSimulator (Li et al., 2018; Li et al., 2020) to evaluate TargetCall. We use four reference genomes to evaluate TargetCall on three different genome sequencing applications. The details of the exact read datasets and reference genomes used to produce all our results can be found in Section 2 of the Supplementary Material.

#### 3.1.10 Evaluation metrics

We evaluate TargetCall using five different metrics: 1) filtering accuracy, 2) basecalling accuracy, 3) relative abundance (RA) estimation accuracy, 4) basecalling execution time, and 5) end-to-end execution time. For the basecalling execution time, we compare the wall-clock time spent on pre-basecalling filtering followed by basecalling of the reads that are accepted by the filter and conventional basecalling. For the end-to-end execution time, we compare the wall-clock time spent on the entire genome analysis pipeline of basecalling, read mapping, and variant calling with and without the use of pre-basecalling filtering. The index generation time of minimap2 is excluded from end-to-end execution time as this is a one-time task per reference genome. When comparing TargetCall with UNCALLED and Sigmap in terms of the end-to-end execution time, we acknowledge that TargetCall benefits from hardware acceleration as it uses GPUs while the other tools use CPUs. Implementing UNCALLED and Sigmap on GPUs could further improve their speed performance.

We evaluate the filtering accuracy of TargetCall by computing its precision and recall. We define precision as the number of reads that TargetCall correctly labels as on-target, divided by the total number of reads that TargetCall labels as on-target. We define recall as the number of reads that TargetCall correctly labels as on-target, divided by the overall number of on-target reads in the dataset. The ground truth on-target reads are determined by the conventional pipeline of basecalling with Bonito and read mapping. An ideal pre-basecalling filter should have 100% recall to maintain accuracy in the downstream analyses and 100% precision to provide the maximum possible runtime performance improvement.

For basecalling accuracy, we use Bonito’s training and evaluation procedure to extract the median identity as basecalling accuracy Bonito (2022). For relative abundance (RA) accuracy, we calculate the difference in relative abundances of viral species after 1) pre-basecalling filtering and 2) conventional basecalling. We compute how much RA deviates from the true RAs after pre-basecalling filtering. RA is defined as the proportion of reads corresponding to a particular species relative to the total number of reads. The true RA refers to the RA obtained after conventional basecalling, which is considered the benchmark. Equation 1 provides the calculation of the deviation in RAs where $TC\text{\_}RA_{i}$ is the RA of species i after TargetCall, and $B\text{\_}RA_{i}$ is the RA of species i after conventional basecalling with Bonito.

$$\text{RA Deviation}=\underset{for\;each\;species\;i}{\sum }100*\frac{\left|TC\text{\_}RA_{i}-B\text{\_}RA_{i}\right|}{B\text{\_}RA_{i}}.$$ (1)

### 3.2 Filtering accuracy

**FIGURE 3:**
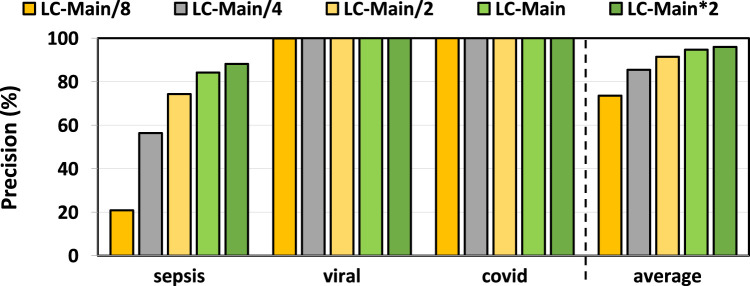
Precision of our evaluated use cases using TargetCall with different LightCall configurations.

**FIGURE 4:**
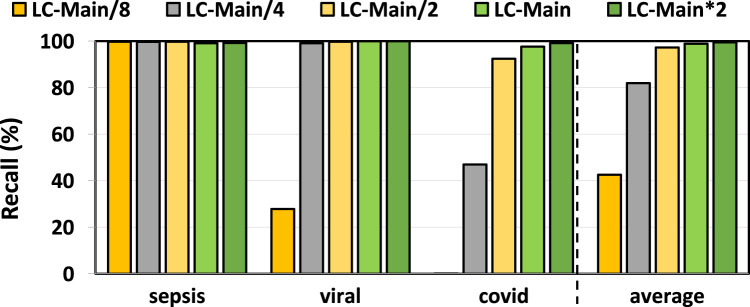
Recall of our evaluated use cases using TargetCall with different LightCall configurations.

In Figures 3, 4, we assess the precision and recall of TargetCall with different LightCall configurations for all use cases explained in Section 3.1. We make four key observations. First, TargetCall’s precision and recall are between 73.59% – 96.03% and 42.57% – 99.45% for different configurations of LightCall on average across all three use cases tested. Second, the precision and recall of TargetCall increases as the model complexity of LightCall increases. Third, increasing the model complexity provides diminishing precision and recall improvements beyond the complexity of the $LC_{\mathrm{Main}}$ model. Fourth, models smaller than $LC_{\mathrm{Main}}$ are sufficient for use cases with small-to-medium target reference sizes, whereas more complex models are required for use cases with large target reference sizes. We conclude that $LC_{\mathrm{Main}*2}$ provides the highest precision and highest recall compared to other LightCall configurations.

### 3.3 Basecalling and relative abundance accuracy

Table 2 shows the basecalling accuracy and the RA deviation from the ground truth relative abundance estimation calculated for each LightCall configuration and using Bonito without pre-basecalling filtering. We evaluate the RA accuracy of TargetCall in the viral detection use case. We use Equation 1 to calculate the RA deviation as the RA accuracy metric.

**TABLE 2:** Basecalling accuracy and relative abundance (RA) deviation.

Model name	Basecalling accuracy (%)	RA deviation (%)
Bonito	94.60	0.00
$LC_{\mathrm{Main\times 2}}$	90.91	0.03
$LC_{\mathrm{Main}}$	89.75	0.08
$LC_{\mathrm{Main∕2}}$	86.83	0.23
$LC_{\mathrm{Main∕4}}$	80.82	0.91
$LC_{\mathrm{Main∕8}}$	70.42	72.19

We make the following two key observations. First, the RA deviation results are negligible ( ≤ 0.1%) for TargetCall configurations with recall higher than 98.5%. The only exception to this observation is our results when using the $LC_{\mathrm{Main∕8}}$ model. Although the basecalling accuracy drop is approximately 10% between $LC_{\mathrm{Main∕8}}$ and $LC_{\mathrm{Main∕4}}$ , the deviation increases substantially because most of the reads cannot be mapped (see the low recall result in Figure 4), which affects the relative abundance estimations. Furthermore, as the read accuracy decreases, the read deviates from its original viral genome while it can still map to other viral genomes. We note that a similar issue is not observed in the precision results of the filtering use case (see Figure 3), as the goal of the filtering is to differentiate a viral genome from a bacterial genome (Section 3.2) rather than correctly mapping a read to its original viral genome compared to other viral genomes.

Second, TargetCall’s minor inaccuracy is not biased toward any specific portion of the target reference. Otherwise, the deviation of the relative abundances would be higher. Losing a small number of on-target reads randomly enables sequencing depth-of-coverage to compensate for the loss of reads. We conclude that TargetCall’s high recall enables accurate estimation for relative abundance calculations.

### 3.4 Basecalling execution time

**FIGURE 5:**
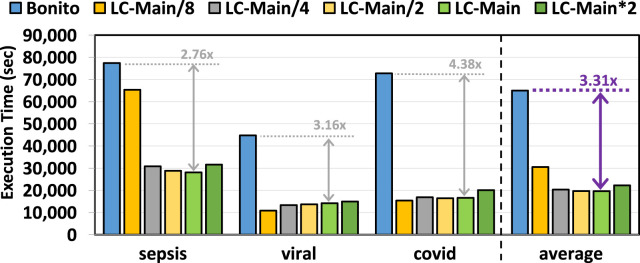
Basecalling execution time of our evaluated use cases using TargetCall with different LightCall configurations.

Figure 5 provides the total execution time of Bonito and Bonito with TargetCall. We make three key observations. First, TargetCall improves the runtime performance of Bonito by 2.13× – 3.31× . Second, both the precision and model complexity of LightCall affect the runtime performance of TargetCall, resulting in a non-linear relationship between model complexity and runtime performance. Precision affects the runtime performance of the filter as lower precision results in a higher number of falsely accepted reads to be basecalled using conventional basecallers. Model complexity affects the runtime performance of the filter as lower model complexity results in higher LightCall runtime performance with lower precision. Third, decreasing the model complexity increases the runtime performance to the point where read filtering accuracy is no longer sufficient to filter out reads correctly. This results in a significant number of reads being falsely accepted by the filter, reducing the runtime performance of TargetCall significantly. We conclude that TargetCall significantly improves the execution time of basecalling.

### 3.5 End-to-end execution time

**FIGURE 6:**
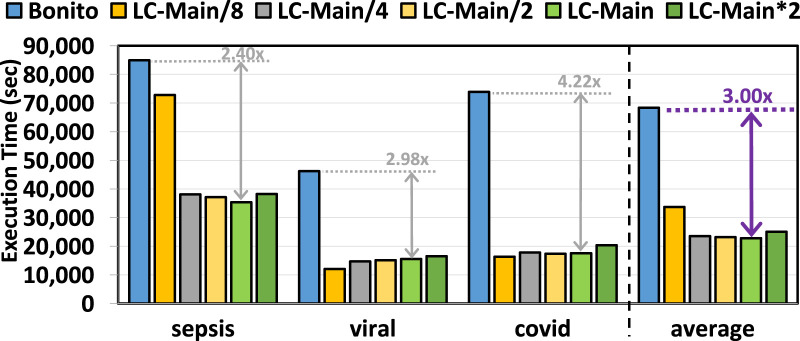
End-to-end execution time of our evaluated use cases using TargetCall with different LightCall configurations.

Figure 6 provides the total time spent on the genome analysis pipeline of basecalling, read mapping, and variant calling with and without the use of pre-basecalling filtering. We used minimap2 (Li, 2018) and DeepVariant (Poplin et al., 2018) for read mapping and variant calling, respectively. We make three key observations. First, TargetCall improves the runtime performance of the entire genome sequence analysis pipeline by 2.03× – 3.00× . Second, the choice of the variant callers affects the end-to-end runtime performance improvement of TargetCall. Because we used a highly accurate neural network-based variant caller, the execution time of variant calling dominated read mapping (not shown). This reduces the end-to-end runtime performance benefits of TargetCall, as the variant calling is performed only on the alignments of on-target reads (i.e., on the reduced dataset) determined during a relatively lightweight read mapping step. Third, similar to basecalling execution time, multiple factors affect the runtime performance of TargetCall with different LightCall configurations, which results in a non-linear relationship between the runtime performance and model complexity of LightCall. We conclude that TargetCall significantly improves the end-to-end execution time of the genome sequence analysis pipeline by providing up to a 3.00× speedup.

### 3.6 Comparison to prior work

We compare TargetCall with the best LightCall configuration ( ${LC}_{Main}$ ) with two state-of-the-art adaptive sampling methods, UNCALLED and Sigmap. Section 3.1 of the Supplementary Material explains the best model selection procedure. We used two different reference genomes for the sepsis use case, but UNCALLED failed to generate the index structure for our default human reference genome (hg38) in a high-end server with 1 TB of main memory. We evaluate only these two methods, as they can readily be repurposed as pre-basecalling filters. The other methods are either not fully open source (Payne et al., 2020) or cannot be repurposed as pre-basecalling filters (Bao et al., 2021).

**FIGURE 7:**
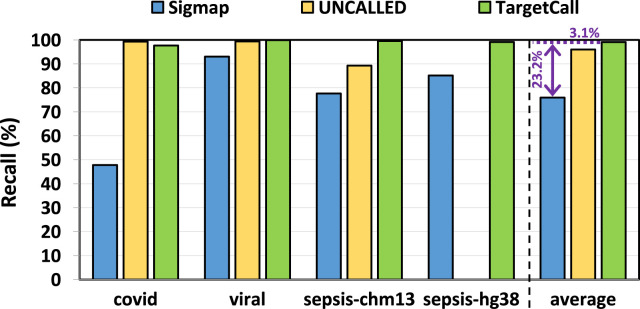
Recall of Sigmap, UNCALLED, and TargetCall.

We compare the recall of TargetCall with that of Sigmap and UNCALLED. The ground truth on-target reads are determined by the conventional pipeline. In our analysis, we observe that UNCALLED could not be executed on the hg38 reference genome. To ensure a fair assessment, we excluded hg38 from the performance evaluation of UNCALLED. Figure 7 shows the recall of Sigmap, UNCALLED, and TargetCall. We make two key observations. First, TargetCall provides significantly higher recall, + 3.09% /+ 23.15% , than UNCALLED/Sigmap on average. Second, TargetCall consistently provides the best recall compared to both methods across all use cases except COVID detection, for which adaptive sampling methods are optimized.

**FIGURE 8:**
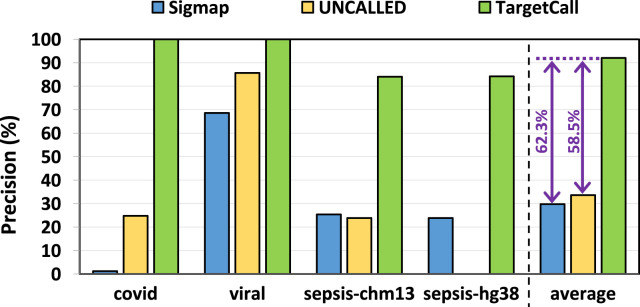
Precision of Sigmap, UNCALLED, and TargetCall.

We compare the precision of TargetCall with that of Sigmap and UNCALLED. Figure 8 shows the precision of Sigmap, UNCALLED, and TargetCall. We make two key observations. First, TargetCall provides significantly higher precision, + 58.48% /+ 62.31% , than UNCALLED/Sigmap on average. Second, TargetCall maintains high precision as the target reference size increases, unlike prior methods. We conclude that TargetCall significantly achieves higher recall and precision in all use cases independent of the target reference size.

**FIGURE 9:**
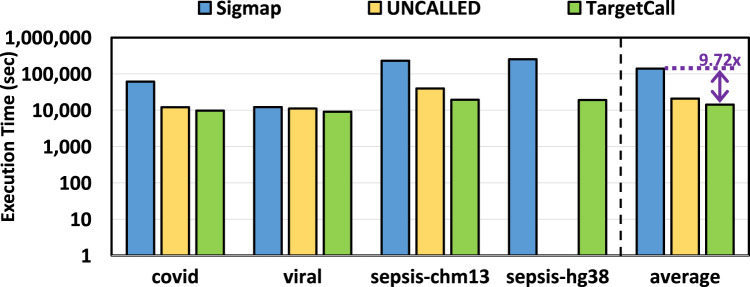
End-to-end execution time of Sigmap, UNCALLED, and TargetCall.

We compare the end-to-end execution time of TargetCall with that of Sigmap and UNCALLED, and the results are shown in Figure 9. We make two key observations. First, we observe that TargetCall outperforms Sigmap by 9.72× and UNCALLED by 1.46× . TargetCall’s higher runtime performance benefits come from its higher precision in filtering out off-target reads. Second, TargetCall’s runtime performance improvements become more significant as the target reference size increases.

**FIGURE 10:**
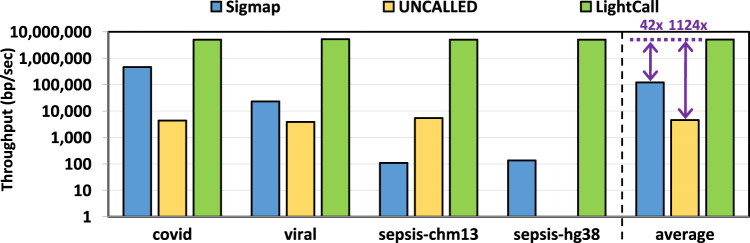
Throughput of Sigmap, UNCALLED, and LightCall.

We evaluate the throughput of TargetCall and compare it with that of Sigmap and UNCALLED. Throughput shows the number of base pairs that the tools process per second. We use uncalled pafstats for evaluating the throughput of UNCALLED and use Sigmap and Bonito output for evaluating the throughput of LightCall. We only evaluate the throughput of the LightCall component of TargetCall, as it has a significantly lower throughput than Similarity Check and is the bottleneck of TargetCall. Figure 10 shows the throughput of Sigmap, UNCALLED, and LightCall. We make three key observations. First, we observe that LightCall improves the throughput of UNCALLED/Sigmap by 1124.03× / 42.08× . Second, we observe that the throughput of LightCall is consistently high, unlike other tools (e.g., Sigmap), whose throughput declines with target reference length. Third, LightCall’s high throughput does not reflect its execution time. The reason for this discrepancy between LightCall’s throughput and execution time is likely because, unlike Sigmap and UNCALLED, TargetCall processes the entire read before labeling it as on-target/off-target. We conclude that TargetCall’s benefits can be amplified by optimizing it further via integrating early filtering of reads that will match/not match the target reference without processing the entire read.

Overall, we conclude that TargetCall 1) significantly improves the end-to-end execution time of basecalling compared to prior methods by filtering out a higher fraction of the off-target reads with its higher precision, 2) improves the recall of prior methods in filtering reads, 3) has a consistently higher throughput than all prior works, and 4) can be accurately applied to target reference lengths that prior methods are unable to be accurately applied while requiring much less (on average 5.76× , see Section 3.2 of Supplementary Material) peak memory than prior works. We believe TargetCall can be used as a lightweight real-time sequence classification and filtering tool due to its high throughput if it is optimized to work with the first few chunks of a read.

### 3.7 TargetCall execution time breakdown

**FIGURE 11:**
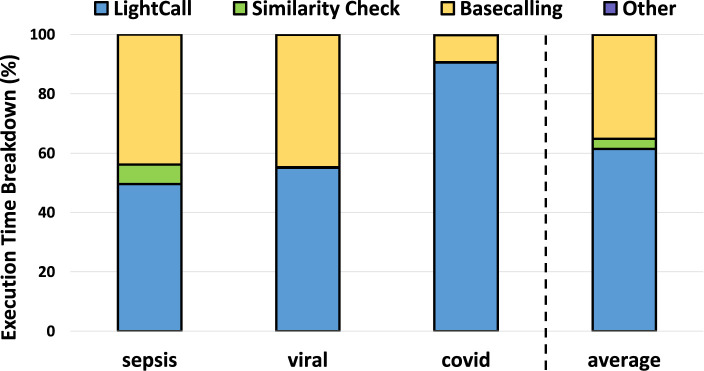
Execution time breakdown of TargetCall.

We analyzed the execution time breakdown of a pipeline that includes TargetCall as the pre-basecalling filter in Figure 11. We make four key observations. First, LightCall is the bottleneck of the new basecalling pipeline that includes pre-basecalling filtering by consuming 61.44% of the total execution time on average. Second, basecalling is still an important computational overhead for the pipeline by consuming 35.04% of the total execution time on average. Third, the computational overhead of basecalling increases with the increased ratio of on-target reads in the dataset, such as in the viral use case. Fourth, the Similarity Check component consumes less than 3.4% of the total execution time on average and does not bottleneck the pipeline that includes TargetCall as the pre-basecalling filter even when used in the expensive alignment mode.

## 4 Discussion

We propose TargetCall, a pre-basecalling filtering mechanism for eliminating the wasted computation in basecalling. TargetCall performs lightweight basecalling to compute noisy reads using LightCall and labels these noisy reads as on-target/off-target using Similarity Check. TargetCall eliminates the wasted computation in basecalling by performing basecalling only on the on-target reads. We focus on convolution-based networks for TargetCall architecture for two reasons: (a) matrix multiplication, the core operation in these networks, is highly suitable for hardware acceleration, facilitating improved performance, and (b) the training and inference of RNN and LSTM models involve sequential computation tasks, which pose significant challenges for acceleration on modern hardware such as GPUs and field-programmable gate arrays (FPGAs) (Singh et al., 2024). We evaluate TargetCall for three different genome sequence analysis use cases of pre-basecalling filtering with varying requirements: covid detection, sepsis detection, and viral detection. We show that TargetCall reduces the execution time of basecalling by filtering out most off-target reads, and it is more applicable than the state-of-the-art adaptive sampling methods. We hope that TargetCall inspires future work in pre-basecalling filtering and real-time sequence classification that accelerate other bioinformatics workloads and emerging applications with its high throughput and recall. We explain future work and optimizations that can build upon TargetCall in Section 4 of the Supplementary Material.

## Data Availability

The data presented in the study are deposited in the Zenodo repository with the following DOI accession numbers: https://zenodo.org/records/7334648 (for D1 part 1), https://zenodo.org/records/7402342 (for D1 part 2), https://zenodo.org/records/7335539 (for D2), https://zenodo.org/records/7335525 (for D3), https://zenodo.org/records/7335517 (for D4), https://zenodo.org/records/7334592 (for D5), and https://zenodo.org/records/7335545 (for all the references we used). Further details about these datasets from D1 to D5 are included in the Supplementary Materials (Supplementary Section 2). TargetCall is fully open source at https://github.com/CMU-SAFARI/TargetCall.

## Footnotes

1 Basecalling accuracy is calculated as the ratio of the number of correctly identified bases to the total number of bases in the read, expressed as a percentage.
