# Supplementary material for: TargetCall: eliminating the wasted computation in basecalling via pre-basecalling filtering
Source: Front Genet. 2024 Oct 28;15:1429306. doi: 10.3389/fgene.2024.1429306 (PMC11551021; doi:10.3389/fgene.2024.1429306)
Supplement: Supplementary file 1 [file DataSheet1.PDF]

Supplementary Material for TargetCall: Eliminating the Wasted Computation in Basecalling via Pre-Basecalling Filtering

Meryem Banu Cavlak, Gagandeep Singh, Mohammed Alser, Can Firtina, Joël Lindegger, Mohammad Sadrosadati, Nika Mansouri Ghiasi, Can Alkan, and Onur Mutlu

1. ADAPTIVE SAMPLING BACKGROUND

Targeted sequencing is a method to selectively sequence reads from the reference genome of interest (i.e., target reference) during sequencing time.[1–3] ONT devices have the potential to enable computational targeted sequencing without the need for library preparation with a feature, known as *Read Until*.[1,4] ONT sequencers that support Read Until can selectively remove a read from the nanopore while the read is being sequenced, called as the adaptive sampling. Read Until can be used to remove off-target reads during sequencing, eliminating wasted computation on off-target reads in basecalling. All adaptive sampling approaches work by labeling a read as on-target or off-target and stopping the sequencing of off-target reads immediately after labeling using Read Until. We discuss three groups of works that perform Read Until, classified based on their methodology to label the read. The first group converts the target reference into a reference raw signal and performs raw signal-level alignment.[4–6] The second group generates noisy sequence representations of the raw signal to compare them with the target reference.[1,7] The third group of works utilize neural network classifiers to label the sequences.[8,9] To our knowledge, none of these works can be fully repurposed as widely-applicable pre-basecalling filters to eliminate the wasted computation in basecalling for a wide range of genome applications as their accuracy drops significantly with the increasing target reference size.

The first group of work compares the raw electrical signals to a target reference without basecalling the signals in two steps.[4] The first step converts the bases of the reference genome into their *synthetic* raw signal representation. The second step identifies similarities between the raw read signal and the synthetic reference signal. Prior work[4] used the Dynamic Time Warping (DTW) algorithm that measures the similarity of a pair of signals to find the similarity. If the read is not similar to target reference, it is labeled as off-target. Unlike TargetCall, this method is not applicable to large target references of millions of base pairs (bp) long due the quadratic time complexity of DTW with respect to the length of the sequences. To address the computational bottleneck of DTW, Sigmap[5] propose to generate an index for the synthetic reference signal. Sigmap queries the generated index as the reads are being sequenced to find the potential similarity positions between the read and the reference signal, avoiding the DTW calculation. These positions refer to matches of short subsequences of the read and the reference signal, and the final labelling is determined based on chaining these match locations. The use of index structure enables Sigmap to be applicable to target references of length up to ∼100 Mbp, however, Sigmap is still significantly less applicable compared to TargetCall. Recently, SquiggleFilter proposed to design an accelerator to make DTW calculation faster[6] but it was unable to make DTW scalable for large target references.

The second group of works is based on converting the raw signal into a set of bases and comparing these bases to the reference to label the raw signal.[1,7] Readfish uses a real-time basecalling method to basecall the read as it is being sequenced and perform read mapping on the basecalled portion of the raw signal.[7] Since basecallers are optimized to work on complete reads, this method results in suboptimal base sequences hence may incorrectly label the reads.[5] To mitigate these drawbacks, UNCALLED uses an index of the reference genome to probabilistically convert the raw signal into a set of short nucleotide subsequences called seeds and cluster them.[1] The read is classified as on-target read if there is a location in the target reference that has significantly more seeds mapping to that region than the others.[1] UNCALLED is also designed to work *only* on small reference genomes with the goal of performing adaptive sampling.[1,5]

The third group of works uses machine learning to label the raw signals in a sample without performing basecalling and costly analyses in the base space.[8,9] SquiggleNet[8] can identify a certain class of species with a

high accuracy where the class membership is determined based on the target reference. However, this approach requires training the machine learning model for each target reference, which cannot be practically applied to classify any type of species due to the high computational costs of training. Therefore, unlike TargetCall, SquiggleNet cannot be used as a widely-applicable pre-basecalling filter. BaseLess[9] utilizes an array of small neural networks to detect a small subsequences from raw signals and match these subsequences with a target reference that share the same subsequence. This design choice provides a flexible solution that can define the set of pre-trained neural network models of subsequences to identify a certain target reference instead of retraining the neural network model for each species. Unfortunately, none of these works can avoid the cost of training the models multiple times (i.e., for each subsequence or species) to identify the target references in raw signals.

Limitations of Adaptive Sampling Approaches. Even though these approaches can discard off-target reads from the genome analysis pipeline hence eliminating the wasted computation in basecalling, all have at least one of the following three key limitations, preventing them to be used as *widely applicable* pre-basecalling filters. First, some[1,5,7] have low (77.5%-90.40%) sensitivity which affects the accuracy of downstream analysis. These tools falsely reject a significant portion (∼10%-∼23%) of the on-target reads. Second, some[1,4–6] are not scalable to the long target references. This happens due to one of the following reasons: 1) use of signal-signal alignment algorithms whose complexity increases linearly with the target reference length,[4,6] 2) use of complex data structures to represent target reference in signal domain that are not scalable to long target reference lengths,[5] and 3) use of probabilistic algorithms that scale poorly with the increasing target reference length.[1] These tools cannot be used for applications that require long target references, such as human reference. Third, some require neural network classifiers to be re-trained for each different application and target reference.[8] The re-training is required since the classifier is trained depending on the specific set of on-target and off-target reads. These approaches cannot be used in a wide range of pre-basecalling filtering applications without significant overheads.

2. EVALUATED DATASETS

Evaluated Read Datasets. We use four real and one simulated dataset to evaluate TargetCall. Table 1 provides details on the evaluated datasets. Datasets D1 & D2, D1 & D4 and D3 & D5 are used for covid detection, sepsis detection and viral detection use cases respectively. For real datasets we randomly sample the datasets provided by prior research to keep a tractable experiment time. We use DeepSimulator to generate the simulated reads.[10,11] We simulate the dataset D5 due to unavailability of open access raw signal files for viral reads.

Table 1. Evaluated read datasets.

List of Read Datasets	Dataset Type	Number of Reads	Source	DOI Accession
(D1) Human	Real	196,000	[12]	10.5281/zenodo.7334648
(D2) SARS-CoV-2	Real	4,000	[13]	10.5281/zenodo.7335539
(D3) Bacterial Mixture (n=7)	Real	72,567	[14]	10.5281/zenodo.7335525
(D4) Bacterial Mixture (n=9)	Real	15,200	[14]	10.5281/zenodo.7335517
(D5) Viral Mixture (n=7)	Simulated	35,000	[10,11]	10.5281/zenodo.7334592

Evaluated Reference Genomes. We use four reference genomes to evaluate TargetCall on three applications. Table 2 lists the details of these reference genomes.

For testing viral detection use case, we used 10 viral reference genomes from NCBI RefSeq (with IDs NC_003977.2, AC_000007.1, NC_009334.1, NC_001526.4, NC_010277.2, NC_001731.1, NC_063383.1, NC_055231.1, NC_045512.2, NC_014361.1); and combined them to use as the target reference.[15] Only 7 of these 10 reference genomes are used for simulating reads of dataset D5*.

*Reference Genomes tested can be accessed with DOI 10.5281/zenodo.7335545

Table 2. List of Reference Genomes Used for each Use Case.

Reference Genome	Reference Length (bp)	Use Case	Datasets Compared	% of Target Reads
Human (GRCh38)	3,088,286,401	Sepsis Detection	D1 & D4	17.64%
Human (Chm13)	3,117,292,070	Sepsis Detection	D1 & D4	17.64%
SARS-CoV-2 (NC_045512.2)	29,903	Covid Detection	D1 & D2	2%
Viral Combined (n=10)	861,552	Viral Detection	D3 & D5	32.51%

3. RESULTS

3.1 Best Model Selection

We evaluate the performance-sensitivity trade-off of TargetCall to determine the best LightCall architecture for TargetCall. Figure 1 aggregates the sensitivity (i.e., recall) and performance improvement (i.e., basecalling speedup) of all LightCall configurations evaluated (except $LC_{Main/8}$). We make the following three key observations. First, LC_{Main} provides the highest (3.31×) speedup in basecalling. Second, LC_{Main*2} provides the highest (99.45%) sensitivity in basecalling. Third, LC_{Main} provides significantly higher (13.36%) speedup than LC_{Main*2} with minimal (0.57%) reductions in recall. Therefore, we select LC_{Main} as the LightCall component of TargetCall. We conclude that TargetCall by using LC_{Main} improves the performance of basecalling by 3.31× by precisely filtering 94.71% of the on-target reads while maintaining high (98.88%) sensitivity in filtering.

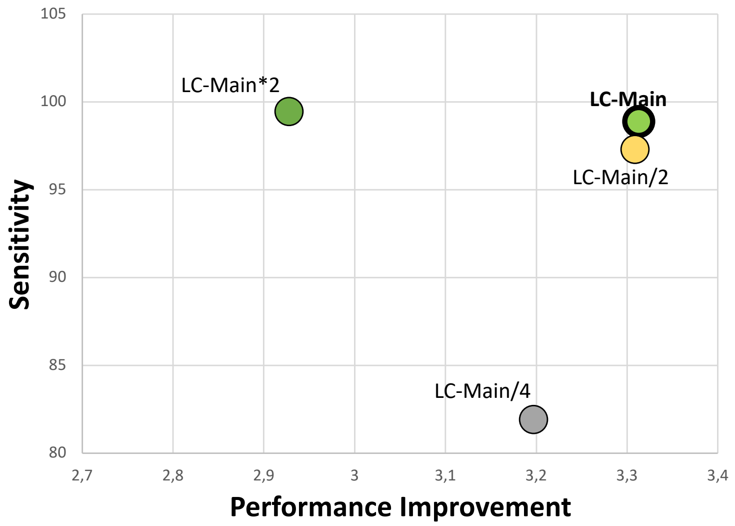

Figure 1. Overall sensitivity (recall) and performance (basecalling speedup) improvement for TargetCall with different LightCall configurations

3.2 Peak Memory

We evaluate the peak memory of TargetCall and compare it against with that of Sigmap and UNCALLED. We subsample 4000 human reads from the dataset to evaluate the peak memory usage of these tools since adding more reads to the experiment does not change the results. We use the linux time command for evaluating the peak memory usage of UNCALLED, Sigmap and Similarity Check. We use the nvidia-smi command to evaluate the peak memory usage of LightCall. The peak memory usage of TargetCall is equal to the maximum peak memory usage of its components: LightCall and Similarity Check. Table 3 demonstrates the peak memory usage of Sigmap, UNCALLED, TargetCall and its components. We make four observations. First, LightCall's peak memory usage is the same across different use cases, since the basecalling algorithm is independent of the target reference. Second, for use cases with small target reference, the peak memory usage of TargetCall is determined

by the peak memory usage of LightCall, whereas its peak memory usage is determined by the peak memory usage of Similarity Check for large target references. Third, TargetCall's peak memory usage is always less than Sigmap's peak memory usage, and it becomes more efficient in terms of peak memory compared to Sigmap as the target reference length increases. Fourth, both UNCALLED's and TargetCall's peak memory usage are low for all use cases ($\leq$ 20GB). We conclude that TargetCall is the only tool that both has low peak memory usage for all target reference lengths and is applicable to all target references.

Table 3. Peak Memory (MB).

**Use Case**	**UNCALLED**	**Sigmap**	**LightCall**	**Similarity Check**	**TargetCall**
Covid	1329.1	3455.0	2005.0	139.6	2005.0
Viral	1385.4	3546.8	2005.0	274.9	2005.0
Sepsis_chm13	8126.4	234504.3	2005.0	16593.9	16593.9
Sepsis_hg38	-	225746.5	2005.0	16974.7	16974.7

4. FUTURE WORK AND OPTIMIZATIONS

The performance and accuracy of TargetCall can be further optimized in three main directions. The first direction is to optimize the LightCall component to achieve higher basecalling accuracy with significant performance improvements. Currently, the LightCall model is designed based on pruning a state-of-the-art basecaller, Bonito's model. The specific model configurations evaluated are chosen based on the insight we developed from prior work. We believe the accuracy and performance of LightCall can be optimized even better with a more methodological approach in specifying the precise configurations, such as neural architecture search.[16]

The second direction is to optimize the Similarity Check component to achieve higher sensitivity, precision and/or performance. Currently, the state-of-the-art read mapper, minimap2, is used as the Similarity Check component of TargetCall. TargetCall's sensitivity can be improved by manually tuning the minimap2 parameters, and its performance can be improved by using it with large window size or without the expensive alignment mode. We left this analysis as part of future work for the following three reasons. First, although the sensitivity and precision of TargetCall can be improved by changing the minimap2 parameters, the TargetCall's precision and recall are already very high (92.1%/99.1% averaged over 4 use cases in Section 3.6). Second, as we show in section 3.7 of the paper that minimap2 consumes less than 3.4% of the execution time of a basecalling pipeline that includes TargetCall as the pre-basecalling filter. Therefore, the performance of TargetCall will not be significantly improved by optimizing the minimap2 parameters. Third, changing the minimap2 parameters will change the reads labeled as on-target, so the reads that needed to be basecalled using state-of-the-art basecallers to evaluate the execution time of TargetCall in addition to its precision/recall. This will increase the experimental time extensively. Therefore, we left this optimization as part of our future work considering the already high recall, precision and performance of TargetCall.

The third direction is to replace the Similarity Check component with a less expensive filter. Read mapping problem aims to match a read to its position in a reference genome. However, the goal of Similarity Check, predicting if a read is coming from a target reference, is simpler than read mapping. Therefore, TargetCall can be optimized by using k-mer or sketch based methods as its Similarity Check component such as KrakenUnique[17] and Mash.[18] Similar to the second direction, we left this as part of future work, as TargetCall is not bottlenecked by its Similarity Check component.

We hope that TargetCall inspires future work in pre-basecalling filtering and raw signal classification that accelerate other bioinformatics workloads and emerging applications with its high throughput and sensitivity. One such application is adaptive sampling where the raw signals are classified in real-time while the reads are being sequenced. We show in Section 3.6 of the paper that TargetCall's throughput is on par with sequencing throughput of ONT devices, and much higher (up to 1124x) than the adaptive sampling approaches we compared against. Further analysis is required to understand to what extent TargetCall can be used for adaptive sampling such as evaluating the sensitivity and precision of TargetCall when only the initial portions of the raw signals are available for classification. We believe the optimizations explained above can be useful for future work that applies TargetCall to adaptive sampling problem.

REFERENCES

[1] S. Kovaka, Y. Fan, B. Ni, W. Timp, and M. C. Schatz, "Targeted Nanopore Sequencing by Real-Time Mapping of Raw Electrical Signal with UNCALLED," *Nature Biotechnology*, 2021.

[2] T. Gilpatrick, I. Lee, J. E. Graham, E. Raimondeau, R. Bowen, A. Heron, B. Downs, S. Sukumar, F. J. Sedlazeck, and W. Timp, "Targeted Nanopore Sequencing with Cas9-Guided Adapter Ligation," *Nature Biotechnology*, 2020.

[3] A. Payne, N. Holmes, T. Clarke, R. Munro, B. Debebe, and M. Loose, "Nanopore Adaptive Sequencing for Mixed Samples, Whole Exome Capture and Targeted Panels," *bioRxiv*, 2020.

[4] M. Loose, S. Malla, and M. Stout, "Real-Time Selective Sequencing Using Nanopore Technology," *Nature Methods*, 2016.

[5] H. Zhang, H. Li, C. Jain, H. Cheng, K. F. Au, H. Li, and S. Aluru, "Real-Time Mapping of Nanopore Raw Signals," *Bioinformatics*, 2021.

[6] T. Dunn, H. Sadasivan, J. Wadden, K. Goliya, K.-Y. Chen, D. Blaauw, R. Das, and S. Narayanasamy, "SquiggleFilter: An Accelerator for Portable Virus Detection," in *MICRO*, 2021.

[7] A. Payne, N. Holmes, T. Clarke, R. Munro, B. J. Debebe, and M. Loose, "Readfish Enables Targeted Nanopore Aequencing of Gigabase-Sized Genomes," *Nat Biotechnol*, 2020.

[8] Y. Bao, J. Wadden, J. R. Erb-Downward, P. Ranjan, W. Zhou, T. L. McDonald, R. E. Mills, A. P. Boyle, R. P. Dickson, D. Blaauw, and J. D. Welch, "SquiggleNet: Real-Time, Direct Classification of Nanopore Signals," *Genome Biology*, 2021.

[9] B. Noordijk, R. Nijland, V. J. Carrion, J. M. Raaijmakers, D. de Ridder, and C. de Lannoy, "baseLess: Lightweight Detection of Sequences in Raw MinION Data," *bioRxiv*, 2022.

[10] Y. Li, R. Han, C. Bi, M. Li, S. Wang, and X. Gao, "DeepSimulator: A Deep Simulator for Nanopore Sequencing," *Bioinformatics*, 2018.

[11] Y. Li, S. Wang, C. Bi, Z. Qiu, M. Li, and X. Gao, "DeepSimulator1. 5: A More Powerful, Quicker and Lighter Simulator for Nanopore Sequencing," *Bioinformatics*, 2020.

[12] J. M. Zook, J. McDaniel, N. D. Olson, J. Wagner, H. Parikh, H. Heaton, S. A. Irvine, L. Trigg, R. Truty, C. Y. McLean, F. M. De La Vega, C. Xiao, S. Sherry, and M. Salit, "An Open Resource for Accurately Benchmarking Small Variant and Reference Calls," *Nat Biotechnol*, 2019.

[13] "Brazil-UK Centre for Arbovirus Discovery, Diagnosis, Genomics and Epidemiology, https://www.caddecentre.org," 2020.

[14] R. R. Wick, L. M. Judd, and K. E. Holt, "Performance of Neural Network Basecalling Tools for Oxford Nanopore Sequencing," *Genome biology*, 2019.

[15] "RefSeq: NCBI Reference Sequence Database, https://www.ncbi.nlm.nih.gov/refseq/."

[16] B. Zoph and Q. V. Le, "Neural Architecture Search with Reinforcement Learning," 2017.

[17] F. P. Breitwieser, D. N. Baker, and S. L. Salzberg, "KrakenUniq: Confident and Fast Metagenomics Classification using Unique k-mer Counts," *Genome Biology*, 2018.

[18] B. D. Ondov, T. J. Treangen, P. Melsted, A. B. Mallonee, S. Bergman, Nicholas H.and Koren, and A. M. Phillippy, "Mash: Fast Genome and Metagenome Distance Estimation using MinHash," *Genome Biology*, 2016.
